# Analysis of predictability of F‐18 fluorodeoxyglucose‐PET/CT in the recurrence of papillary thyroid carcinoma

**DOI:** 10.1002/cam4.867

**Published:** 2016-08-19

**Authors:** Suk Kyeong Kim, Young So, Hyun Woo Chung, Young Bum Yoo, Kyung Sik Park, Tae Sook Hwang, Bokyung Kim, Won Woo Lee

**Affiliations:** ^1^ Department of Internal MedicineKonkuk University School of MedicineSeoulKorea; ^2^ Department of Nuclear MedicineKonkuk University School of MedicineSeoulKorea; ^3^ Bioimaging Translational Open Innovation CenterKonkuk University School of MedicineSeoulKorea; ^4^ Department of SurgeryKonkuk University School of MedicineSeoulKorea; ^5^ Department of PathologyKonkuk University School of MedicineSeoulKorea; ^6^ Department of PhysiologyKonkuk University School of MedicineSeoulKorea; ^7^ Department of Nuclear MedicineSeoul National University College of MedicineSeoulKorea

**Keywords:** F‐18 fluorodeoxyglucose‐positron emission tomography/computed tomography, lymph node metastasis, papillary thyroid carcinoma, prognosis, recurrence

## Abstract

Whether preoperative F‐18 fluorodeoxyglucose (FDG)‐positron emission tomography/computed tomography (PET/CT) can predict recurrence of papillary thyroid carcinoma (PTC) remains unclear. Herein, we evaluated the potential of primary tumor FDG avidity for the prediction of tumor recurrence in PTC patients. A total of 412 PTC patients (72 males, 340 females; age: 47.2 ± 12.2 years; range: 17–84 years) who underwent FDG‐PET/CT prior to total thyroidectomy (*n* = 350), subtotal thyroidectomy (*n* = 2), or lobectomy (*n* = 60) from 2007 to 2011 were analyzed. The predictive ability for recurrence was investigated among various clinicopathological factors, BRAF^V^ ^600E^ mutation, and preoperative FDG avidity of the primary tumor using Kaplan–Meier (univariate) and Cox proportional hazards regression (multivariate) analyses. Of the 412 patients, 19 (4.6%) experienced recurrence, which was confirmed either by pathology (*n* = 17) or high serum thyroglobulin level (*n* = 2), during a mean follow‐up period of 43.9 ± 16.6 months. Of the 412 patients, 237 (57.5%) had FDG‐avid tumors (maximum standardized uptake value, 7.1 ± 7.0; range: 1.6–50.5). Kaplan–Meier analysis revealed that tumor size (*P* = 0.0054), FDG avidity of the tumor (*P* = 0.0049), extrathyroidal extension (*P* = 0.0212), and lymph node (LN) stage (*P* < 0.0001) were significant predictors for recurrence. However, only LN stage remained a significant predictor in the multivariate analysis (*P* < 0.0001). Patients with FDG‐avid tumors had higher LN stage (*P* < 0.0001), larger tumor size (*P* < 0.0001), and more frequent extrathyroidal extension (*P* < 0.0001). In conclusion, FDG avidity of the primary tumor in preoperative FDG‐PET/CT could not predict the recurrence of PTC. LN stage was the only identified predictor of PTC recurrence.

## Introduction

In differentiated thyroid carcinoma patients, the role of F‐18 fluorodeoxyglucose (FDG)‐positron emission tomography/computed tomography (PET/CT) has been limited primarily to postoperative surveillance. FDG‐PET/CT is known to be useful in the follow‐up of differentiated thyroid carcinoma patients with elevated serum thyroglobulin levels and negative radioiodine whole body scans 1, 2. Furthermore, some reports have shown that FDG‐PET/CT could change the treatment plan in postoperative differentiated thyroid carcinoma patients 3, 4, and that the prognosis of metastatic thyroid carcinoma in the postoperative setting could be effectively predicted using preoperative FDG‐PET/CT 5, 6.

However, the role of preoperative FDG‐PET/CT in differentiated thyroid carcinoma has not yet been established, and FDG‐PET/CT is currently not recommended in the preoperative work‐up 7, owing to the fact that it does not appear to provide significant additional information on the T and N stages 8, 9 or supplemental information on the differential diagnosis of indeterminate thyroid nodules in these patients 10, 11. On the contrary, a small number of studies have reported that preoperative FDG‐PET/CT could reduce unnecessary surgery in patients with indeterminate thyroid nodules 12, 13. Approximately one third of all incidentally detected thyroid nodules on FDG‐PET/CT was malignant, thus requiring further evaluation 14, 15. Furthermore, the FDG avidity of differentiated thyroid carcinoma has been shown to be associated with tumor size, lymph node (LN) metastasis, extrathyroidal extension, and lymphovascular invasion 16, 17, and these factors are known to be associated with a poor prognosis 9, 18. Therefore, it can be speculated that preoperative FDG‐PET/CT may be useful for the prediction of the prognosis of differentiated thyroid carcinoma.

In this study, we evaluated whether FDG avidity of the primary tumor upon preoperative FDG‐PET/CT is a predictive factor for the recurrence of papillary thyroid carcinoma (PTC).

## Materials and Methods

### Patients

This study was approved by the Institutional Review Board. Patient consent was not required in this study. The records of patients with newly diagnosed PTC who underwent FDG‐PET/CT prior to thyroid surgery at our institution from January 2007 to December 2011 were reviewed. We identified a total of 434 patients with PTC who underwent FDG‐PET/CT within 3 months prior to operation. Of these 434 patients, 4 patients with initial distant metastases (3 lung metastases, 1 bone metastasis) were excluded. Eighteen patients who showed diffuse hypermetabolism at the thyroid gland on preoperative FDG‐PET/CT were also excluded, since their primary tumor sites in the thyroid gland were difficult to determine. Therefore, a total of 412 PTC patients who underwent FDG‐PET/CT prior to operation were finally enrolled in this study.

### FDG‐PET/CT scan procedure

All patients fasted for at least 6 h before FDG (4.8 MBq/kg) were intravenously injected in the resting state. An intravenous CT contrast agent was not administered in this study. The blood glucose level was checked in all patients and was lower than 120 and 200 mg/dL for nondiabetic and diabetic patients, respectively. PET/CT images were acquired 60 min after FDG injection with a GEMINI scanner (Philips Medical System, Cleveland, OH), with the patient positioned with both arms down. The CT scan comprised dual slice CT. The scan field of view was from the skull base to the mid‐thigh level. The CT scan was performed using a standardized protocol of 120 kV X‐ray voltage, 50 mA tube current, a 0.75‐sec tube rotation time per rotation, 1.5 pitch, and a section thickness of 5 mm. Immediately after the CT scan, PET images were acquired using a conventional three‐dimensional protocol with 2.5 min per frame.

### Interpretation and analysis of FDG‐PET/CT scan

Two nuclear medicine physicians with more than 10 years experience assessed the FDG‐PET/CT images. FDG uptake in PTCs was categorized as FDG‐avid tumor if there was a focal discrete FDG uptake in the thyroid that corresponds to the location recorded in pathological reports. If there was no discernible FDG uptake higher than surrounding thyroid tissue, it was categorized as non‐FDG‐avid tumor. In the case of multifocal tumors, the largest tumor was selected for interpretation. For FDG‐avid tumors, the maximum standardized uptake value (SUVmax) was calculated. The SUV was defined as the decay‐corrected radioactivity per unit volume divided by the injected radioactivity per body weight of the patient.

### Treatment and follow‐up of patients

Thyroid surgery was performed within 3 months of FDG‐PET/CT. Of the total 412 patients, 350 patients underwent total thyroidectomy, 2 patients underwent subtotal thyroidectomy, and the remaining 60 patients underwent lobectomy. Cervical LN dissection was performed in 375 (91.0%) patients, and the mean number of dissected LNs was 13.2 ± 13.0 (range: 1–95). The BRAF^V600E^ mutation status was evaluated in 301 patients (73.1%) by the pyrosequencing method, as described previously 19.

Radioactive iodine ablation therapy was performed in 302 patients; 71 patients received low‐dose radioactive iodine ablation therapy (1.11 GBq), while the remaining 231 patients received high‐dose radioactive iodine ablation therapy (up to 7.4 GBq).

The patients were followed up by serum thyroglobulin (immunoradiometric assay kit; ZenTech, Angleur, Belgium) and thyroglobulin antibody (radioimmunoassay kit; BRAHMS, Henningsdorf, Germany) level evaluations, radioiodine whole body scan, thyroid ultrasonography, chest posteroanterior radiography, chest CT, and FDG‐PET/CT. The mean follow‐up period was 43.9 ± 16.6 months (range: 1.9–87.0 months).

### Statistical analysis

Statistical analyses were performed to determine the predictive factors for recurrence among tumor size (microcarcinoma vs. macrocarcinoma), FDG avidity of the primary tumor, multifocality of the primary tumor, extrathyroidal extension (none vs. microscopic vs. macroscopic), LN stage (pN0 vs. pN1a vs. pN1b), sex, age (<45 vs. ≥45 years), and BRAF^V600E^ mutation status. First, univariate analyses were performed using the Kaplan–Meier method, with the significance of the differences between the disease‐free survival curves tested using the log‐rank (Mantel–Cox) test. Next, multivariate disease‐free survival analysis for independent prognostic factors was performed using a Cox proportional hazards model with the significant univariate variables. Finally, the differences between FDG‐avid and non‐FDG‐avid tumors were analyzed using either the independent sample *t*‐test (tumor size, age) or chi‐squared test (multifocality of the primary tumor, extrathyroidal extension, LN stage, sex, and BRAF^V600E^ mutation status). MedCalc version 15.2.2 (MedCalc Software bvba, Ostend, Belgium) was used for the analyses. *P* < 0.05 was considered to be statistically significant.

## Results

### Patient characteristics

The clinicopathological characteristics of the 412 PTC patients are summarized in Table 1.

**Table 1:** Clinicopathological characteristics of the 412 papillary thyroid carcinoma patients

Characteristic	Value
Sex (male:female)	72:340
Mean age (range), years	47.2 ± 12.2 (17–84)
Operation (TT:STT:L)	350:2:60
Mean primary tumor size (range), cm	1.07 ± 0.74 (0.20–5.0)
Unifocal tumor:multifocal tumor	271:141
Extrathyroidal extension (none:microscopic:macroscopic)	246:152:14
Lymph node stage (pN0:pN1a:pN1b)	214:121:40
BRAF^V600E^ mutation (+:−)	277:24
RAI ablation (none:low:high)	110:71:231
FDG‐avid:Non‐FDG‐avid tumor	237:175

TT, total thyroidectomy; STT, subtotal thyroidectomy; L, lobectomy; RAI, radioactive iodine; FDG, fluorodeoxyglucose.

The PTC subtypes were classical, follicular variant, squamous metaplasia, tall cell variant, oncocytic variant, and Warthin‐like type in 385 (93.4%), 20 (4.9%), 4 (0.97%), 1 (0.24%), 1, and 1 patients, respectively. Thirty patients had other malignancies, including breast cancer, stomach cancer, colon cancer, lung cancer, hepatocellular carcinoma, ovarian cancer, malignant melanoma, and invasive thymoma in 26, 7, 2, 1, 1, 1, 1, and 1 patients, respectively, whereas there was no case of head and neck cancer. Of all 412 patients, 255 (61.9%) patients had microcarcinoma (≤1 cm), while the remaining 157 (38.1%) patients had macrocarcinoma (>1 cm).

The primary tumor sites were visible on FDG‐PET/CT in 237 (57.5%) patients, and their mean SUVmax was 7.1 ± 7.0 (range: 1.6–50.5). The clinicopathological characteristics were compared between FDG‐avid and non‐FDG‐avid tumors (Table 2). The size of FDG‐avid tumors was greater than that of non‐FDG‐avid tumors (1.39 ± 0.79 vs. 0.64 ± 0.32 cm, *P* < 0.0001). Extrathyroid extension was more frequently observed in FDG‐avid tumors (none:microscopic:macroscopic = 116:107:14) compared to in non‐FDG‐avid tumors (none:microscopic:macroscopic = 130:45:0, *P* < 0.0001). Furthermore, LN stage was higher in FDG‐avid than non‐FDG‐avid patients (*P* < 0.0001) (Table 2). However, the multifocality of the primary tumor (*P* = 0.4483), sex (*P* = 0.5847), age (*P* = 0.2151), and BRAF^V600E^ mutation status (*P* = 0.0700) did not significantly differ according to the FDG avidity of the primary tumor (Table 2).

**Table 2:** Comparison of clinicopathological characteristics between FDG‐avid and non‐FDG‐avid tumors

	FDG‐avid tumors (*n* = 237)	Non‐FDG‐avid tumors (*n* = 175)	*P* value
Sex (male:female)	44:193	28:147	0.5847
Age (years)	47.9 ± 13.2	46.4 ± 10.9	0.2151
Operation (TT:STT:L)	216:1:20	134:1:40	0.0002
Primary tumor size (cm)	1.39 ± 0.79	0.64 ± 0.32	<0.0001
Unifocal:multifocal tumor	160:77	111:64	0.4483
Extrathyroidal extension (none:microscopic:macroscopic)	116:107:14	130:45:0	<0.0001
Lymph node stage (pN0:pN1a:pN1b)	108:76:37	106:45:3	<0.0001
BRAF^V600E^ mutation (+/−)	163/9	114/15	0.0700
RAI ablation (none:low:high)	43:29:165	67:42:66	<0.0001
Recurrence (+:−)	17:220	2:173	0.0077

FDG, fluorodeoxyglucose; TT, total thyroidectomy; STT, subtotal thyroidectomy; L, lobectomy; RAI, radioactive iodine.

Of the 412 patients, 19 (4.6%) experienced recurrence during the follow‐up. The recurrence was confirmed by either pathology of dissected cervical LNs (*n* = 17) or by a high serum thyroglobulin level (*n* = 2). Table 3 shows a comparison of the clinicopathological characteristics of recurred versus not recurred PTC patients.

**Table 3:** Comparison of clinicopathological characteristics between recurred and not recurred papillary thyroid carcinoma patients

	Recurred (*n* = 19)	Not recurred (*n* = 393)	*P* value
Sex (male:female)	5:14	67:326	0.4656
Age (years)	48.2 ± 15.5	47.2 ± 12.1	0.7208
Operation (TT:STT:L)	332:2:59	18:0:1	0.4714
Primary tumor size (cm)	1.60 ± 0.98	1.05 ± 0.71	0.0015
Unifocal:multifocal tumor	10:9	261:132	0.3227
Extrathyroidal extension (none:microscopic:macroscopic)	6:11:2	240:141:12	0.0183
Lymph node stage (pN0:pN1a:pN1b)	1:6:10	213:115:30	<0.0001
BRAF^V600E^ mutation (+:−)	11:0	266:24	0.6689
RAI ablation (none:low:high)	2:0:17	108:71:214	0.0093
FDG‐avid:Non‐FDG‐avid tumor	17:2	220:173	0.0077
SUVmax of FDG‐avid tumors	7.56 ± 6.79	7.08 ± 7.04	0.7847

TT, total thyroidectomy; STT, subtotal thyroidectomy; L, lobectomy; RAI, radioactive iodine; FDG, fluorodeoxyglucose; SUVmax, maximum standardized uptake value.

### Univariate analysis (Kaplan–Meier method)

**Figure 1:**
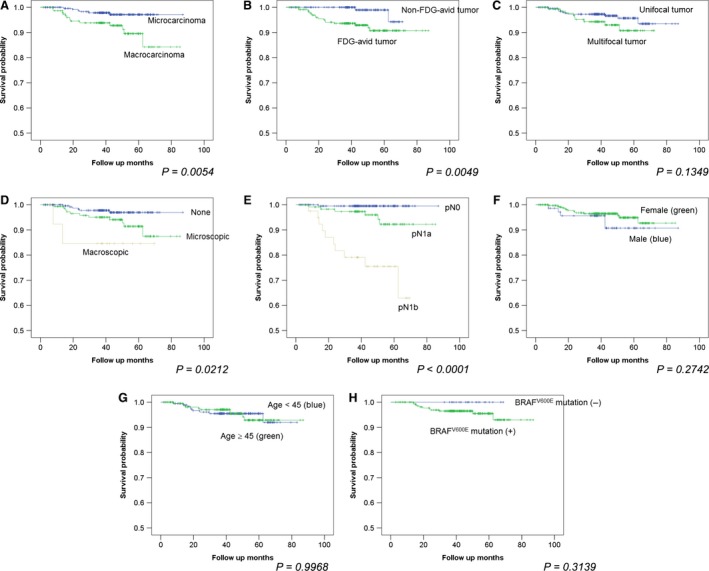
Kaplan–Meier survival analysis results for largest primary tumor size (A), fluorodeoxyglucose (FDG) avidity of the primary tumor (B), multifocality (C), extrathyroidal extension (none vs. microscopic vs. macroscopic) (D), LN stage (pN0 vs. pN1a vs. pN1b) (E), sex (F), age (G), and BRAF^V^ ^600E^ mutation status (H).

In the Kaplan–Meier analyses, tumor size (*P* = 0.0054), FDG avidity of the primary tumor (*P* = 0.0049), extrathyroidal extension (*P* = 0.0212), and LN stage (*P* < 0.0001) were found to be significant predictors of recurrence. However, multifocality of the primary tumor (*P* = 0.1349), sex (*P* = 0.2742), age (<45 vs. ≥45 years) (*P* = 0.9968), and BRAF^V600E^ mutation status (*P* = 0.3139) were not significant predictors of recurrence (Table 4 and Fig. 1).

**Table 4:** Univariate analysis results

Variable	Hazard ratio	95% confidence interval	*P* value
Tumor size	3.6051	1.4244–9.1245	0.0054
FDG avidity	6.2554	2.5182–15.5389	0.0049
Multiplicity	1.9577	0.7436–5.1544	0.1349
Extrathyroidal extension (none vs. microscopic vs. macroscopic)	2.6130	1.2691–5.3799	0.0212
LN stage (pN0 vs. pN1a vs. pN1b)	6.3677	3.0470–13.3074	<0.0001
Sex	1.7542	0.5287–5.8211	0.2742
Age	1.0018	0.4071–2.4656	0.9968
BRAF^V600E^ mutation	ND	ND	0.3139

FDG, fluorodeoxyglucose; ND, not determined; LN, lymph node.

### Multivariate analysis (Cox proportional hazards model)

In the Cox proportional hazards model, including significant univariate variables (tumor size, FDG avidity of the primary tumor, extrathyroidal extension, and LN stage), only LN stage was found to be a significant predictive factor of recurrence (*P* < 0.0001, hazard ratio 6.3677, 95% confidence interval 3.0470–13.3074).

## Discussion

In the current study, preoperative FDG‐PET/CT was not proven to be useful for the prediction of PTC recurrence. However, the FDG avidity of the primary tumor was found to be highly associated with known prognostic indicators, including LN stage, the only independent predictor in this study. The relatively short follow‐up period (mean: 44 months) and high proportion of microcarcinomas (61.9%) may have led to the low recurrent events (4.6%) compared to other studies. If we could have followed the patients for longer and enrolled more macrocarcinoma patients, more patients would likely have experienced recurrence, and FDG‐PET/CT might have been shown to be a significant predictor for the recurrence of PTC. As LN stage remained the only significant predictor in the multivariate analysis, this indicates that LN stage has stronger statistical power than FDG‐PET/CT for the prediction of short‐term PTC recurrence.

There are two major reasons for the inclusion of such a high number of microcarcinomas in the current study. First, overdiagnosis of PTC by routine thyroid ultrasonography, and second, too generous coverage of FDG‐PET/CT in our country. In South Korea, many health promotion centers have included thyroid ultrasonography in the routine health check program, and this has dramatically increased the detection rate of early differentiated thyroid carcinomas 20. Furthermore, many patients with solid tumors, including differentiated thyroid carcinomas, have been able to receive insurance coverage from the national health insurance system for FDG‐PET/CT from 2006. Since then, FDG‐PET/CT scans have been widely performed in differentiated thyroid carcinoma patients before surgery, even in microcarcinoma patients. However, recently, the policy has been changed not to cover preoperative FDG‐PET/CT in differentiated thyroid carcinoma.

The patient inclusion criteria in the current study are another issue. In our study, lobectomy was performed in 60 patients with microcarcinomas. Although the extent of surgery is well known to affect recurrence and survival of PTC, this does not apply to microcarcinomas 21. However, when we analyzed the remaining 352 patients after excluding the 60 patients who underwent lobectomy, similar results were obtained (data not shown).

Recently, Piccardo et al. reported on 54 FDG‐avid primary differentiated thyroid carcinoma patients incidentally detected on FDG‐PET/CT. The authors reported that the FDG avidity of the primary differentiated thyroid carcinoma was associated with the persistence or progression of the disease, although, on multivariate analysis, the FDG avidity did not add further prognostic information 22. Unlike the report of Piccardo et al., our study enrolled both FDG‐avid and non‐FDG‐avid tumors, and we evaluated only PTCs in a larger number of patients, thus enhancing the integrity of our study.

Except for the FDG avidity of the primary tumor and BRAF^V600E^ mutation status, all other clinicopathological prognostic factors analyzed (tumor size, multifocality of the primary tumor, extrathyroid extension, LN stage, sex, and age) are well‐known prognostic factors for PTC. However, in the univariate analyses of our study, only the tumor size, FDG avidity of the primary tumor, extrathyroid extension, and LN stage were found to be significant predictors of recurrence, while the multifocality of the primary tumor, sex, age, and BRAF^V600E^ mutation status were not. This result is partly backed up by the findings of a recent systemic review and meta‐analysis of 13 original articles published from 2005 to 2014 on the risk factors influencing the recurrence of PTC 23. In this review, age and multifocality were not significantly correlated with recurrence, in contrast to sex, extrathyroid extension, LN stage, tumor size, distant metastasis, thyroid surgery type, and I–131 therapy.

Finally, there are a number of studies that have shown the correlation between BRAF^V600E^ mutation and poor prognosis of PTC 24, with a recent study reporting a correlation between BRAF^V600E^ mutation and poor prognosis of papillary thyroid microcarcinomas 25. In our study, among the 301 patients subjected to BRAF^V600E^ mutation status analysis, 277 (92.0%) patients showed a positive result (Table 1). This high proportion of positive test results may have influenced the statistical analysis, since the hazard ratio and its 95% confidence interval could not be determined in the univariate analysis (Table 4). Hence, we consider that a large‐scale study is needed in the future to explain this result.

In conclusion, although FDG avidity of primary tumor in preoperative FDG‐PET/CT correlated with clinicopathological parameters, including LN stage, it could not predict the recurrence of PTC independently. LN stage was the only identified predictor of PTC recurrence.

## Conflict of Interest

There is no conflict of interest to declare.
